# Genetic polymorphisms in the CD14 gene are associated with monocyte activation and carotid intima-media thickness in HIV-infected patients on antiretroviral therapy

**DOI:** 10.1097/MD.0000000000004477

**Published:** 2016-08-07

**Authors:** Yean K. Yong, Esaki M. Shankar, Clare L.V. Westhorpe, Anna Maisa, Tim Spelman, Adeeba Kamarulzaman, Suzanne M. Crowe, Sharon R. Lewin

**Affiliations:** a Centre of Excellence for Research in AIDS (CERiA); b Tropical Infectious Diseases Research and Education Centre (TIDREC), Department of Medical Microbiology, Faculty of Medicine, University of Malaya, Kuala Lumpur, Malaysia; c Centre for Biomedical Research; d Centre for Population Health, Burnet Institute; e Department of Infectious Diseases, Alfred Hospital and Monash University, Melbourne, Australia; f Infectious Disease Unit, University Malaya Medical Centre, Kuala Lumpur, Malaysia; g Peter Doherty Institute for Infection and Immunity, The University of Melbourne, Melbourne, Australia; h Division of Infection Biology and Microbiology, Department of Life Sciences, School of Basic and Applied Sciences, Central University of Tamil Nadu (CUTN), Neelakudi Campus, Tiruvarur, India.

**Keywords:** atherosclerosis, CD14, cIMT, HIV infection, monocytes, sCD14, sCD163, SNPs, TLR4

## Abstract

HIV-infected individuals on antiretroviral therapy (ART) are at increased risk of cardiovascular disease (CVD). Given the relationship between innate immune activation and CVD, we investigated the association of single-nucleotide polymorphisms (SNPs) in *TLR4* and *CD14* and carotid intima-media thickness (cIMT), a surrogate measurement for CVD, in HIV-infected individuals on ART and HIV-uninfected controls as a cross-sectional, case-control study. We quantified the frequency of monocyte subsets (CD14, CD16), markers of monocyte activation (CD38, HLA-DR), and endothelial adhesion (CCR2, CX3CR1, CD11b) by flow cytometry. Plasma levels of lipopolysaccharide, sCD163, sCD14, sCX3CL1, and sCCL2, were measured by ELISA. Genotyping of *TLR4* and *CD14* SNPs was also performed. The TT genotype for *CD14*/−260SNP but not the CC/CT genotype was associated with elevated plasma sCD14, and increased frequency of CD11b+CD14+ monocytes in HIV-infected individuals. The TT genotype was associated with lower cIMT in HIV-infected patients (n = 47) but not in HIV-uninfected controls (n = 37). The AG genotype for *TLR4*/*+896* was associated with increased CX3CR1 expression on total monocytes among HIV-infected individuals and increased sCCL2 and fibrinogen levels in HIV-uninfected controls. SNPs in *CD14*/−*260* and *TLR4*/*+896* were significantly associated with different markers of systemic and monocyte activation and cIMT that differed between HIV-infected participants on ART and HIV-uninfected controls. Further investigation on the relationship of these SNPs with a clinical endpoint of CVD is warranted in HIV-infected patients on ART.

## Abstract 2

Supplemental Digital Content is available in the text

## 1 Introduction

Human immunodeficiency virus (HIV)-infected individuals on antiretroviral therapy (ART) have a 1.5- to 2-fold increased risk for development of cardiovascular disease (CVD) compared with uninfected individuals.^[1]^ Aside from traditional cardiovascular risk factors, other predisposing factors for CVD in this population include lipodystrophy,^[2]^ long-term use of protease inhibitors,^[3]^ and abacavir.^[4]^ However, very few studies have investigated the role of genetic determinants of inflammation and CVD in HIV-infected individuals.

Multiple studies have shown an association between atherosclerosis and other serious non-AIDS-events are associated with chronic immune activation.^[5,6]^ The cause of immune activation is multifactorial and includes HIV-induced damage to the gastrointestinal tract and persistent microbial translocation. Clinical studies in HIV-uninfected individuals support the link between microbial translocation, measured as circulating lipopolysaccharide (LPS) in blood, and development of atherosclerosis and metabolic complications.^[7,8]^

LPS binds to both CD14 and TLR4 on the surface of monocytes/macrophages to augment the inflammatory response (reviewed in^[9]^), and single-nucleotide polymorphisms (SNP) in each of these genes can lead to differential response of these specific receptors on monocytes/macrophages.^[10,11]^ In the human TLR4 gene, +*896 A*>*G* (rs4986790) and *+1196 C*>*T* (rs4986791) are 2 common SNPs (15% and 10% respectively)^[12]^ that are cosegregated in >95% of the Caucasian population.^[13]^ Both SNPs are located in exon 3 of *TLR4* and confer an aminoacid change at Asp299Gly and Thr399Ile, respectively, affecting the extracellular domain of TLR4.^[14]^ A recent study suggested that Asp299Gly may interfere with dimerization of TLR4, which subsequently prevents the recruitment of myeloid differentiation primary response 88 (MyD88) and TIR-domain-containing adapter-inducing interferon (TRIF) molecules to the intracellular docking domain of TLR4 with subsequent impairment of LPS signaling.^[15]^ Some studies indicate that individuals heterozygous for both SNPs have reduced airway responsiveness to inhaled LPS derived from *Escherichia coli* suggesting that the phenotype could protect against LPS-induced asthma.^[16]^

The other common polymorphism known to occur that would affect LPS-mediated inflammation is in the CD14 gene. The *CD14*/−260 C>T (also called −159 C>T) has been reported in ∼48% of Caucasians and has been associated with the differential expression of both membrane (m) and soluble (s) forms of CD14.^[10]^ Using transfection and reporter assays, it has been shown that this SNP can enhance gene promoter activities.^[17,18]^ Further studies have shown that this SNP in the *CD14* promoter region is located near the binding domain of the suppressor protein (Sp) transcription factor. Therefore, the C>T SNP at this locus can lead to reduced binding of Sp to its DNA domain eventually enhancing the expression of CD14.^[17,19]^ This proposed functional effect is consistent with findings of higher levels of circulating sCD14^[10]^ as well as higher density of membrane-bound CD14 in carriers of T versus C alleles.^[10]^ Additionally, increased sCD14 and TNF-α production following LPS-stimulation of whole blood has been reported in carriers of T versus C alleles.^[10]^

Prior genotype-phenotype investigations in HIV-uninfected patients have shown an association between the *CD14*/−*260* and an increased risk of myocardial infarction^[20]^ and the occurrence of larger coronary plaque volume in individuals with stable coronary artery disease.^[21]^ However, other studies have not confirmed this association.^[22]^ Elevated plasma levels of highly sensitive C-reactive protein (hs-CRP)^[23]^ and IL-6 levels^[24]^ as well as sCD163, a monocyte activation marker,^[25]^ have also been reported in TT homozygotes as compared to CC carriers.^[25]^ These data collectively suggest that the *CD14*/−*260* SNP may alter the expression of CD14 and potentially influence the synthesis of proinflammatory mediators upon stimulation with LPS, which in turn could influence the onset and progression of atherosclerosis.

We have recently investigated the relationship between *TLR4*/*+896* A>G (rs4986790), *+1196* C>T (rs4986791), and *CD14*/−260 C>T (rs2569190) SNPs, and the expression of inflammatory markers on monocytes and soluble activation markers in plasma from a cohort of HIV-infected participants on ART in Malaysia. These individuals had a low risk for development of CVD with a median 10-year Framingham score of 5%.^[25]^ We found that *CD14*/−260 SNP was associated with increased monocyte activation but not with carotid artery intima-media thickness (cIMT), a measurement of thickening of blood vessels.^[25]^ Here, we extend these studies using a Caucasian cohortto determine the association of these putative SNPs with subclinical atherosclerosis measured as cIMT and markers of monocyte activation.

## 2 Materials and methods

### 2.1 Cohort description and parameters

The current investigation is a substudy of a previously reported observational study (HIV and Cardiovascular Health, HaCH) of monocyte activation and subclinical atherosclerosis in HIV-infected subjects on ART.^[26]^ This cross-sectional case-control study included 47 HIV-infected patients receiving ART and 37 HIV-negative controls. HIV-infected patients receiving ART were recruited from the Department of Infectious Diseases at the Alfred Hospital in Melbourne, Australia. HIV-uninfected controls with comparable demography were recruited from among the general public via advertisement. Inclusion and exclusion criteria have been previously reported.^[26]^ Patients receiving protease inhibitors within the last 6 months, receiving statins (also applicable to HIV-negative controls) or with HIV viremia (>50 RNA copies/mL) were excluded.

All participants were assessed for visual evidence of subclinical atherosclerotic lesions in the carotid artery by ultrasonographic investigations. Atherosclerosis was measured using cIMT, by taking the median of 12 cIMT measurements of the distal wall of the common carotid arteries within 2 cm of the carotid bifurcation (6 measurements of each artery). Subclinical atherosclerosis was defined as cIMT >0.7 mm. The risk factors associated with development of CVD risk factors were recorded as previously described^[26]^ and CVD risk were calculated using the Framingham risk score (a composite index that comprise of age, gender, total cholesterol, HDL, systolic BP, and smoking).^[27]^ Racial information was not collected. All plasma and PBMC samples from HIV-infected individuals and healthy controls were de-identified upon collection and processed by standard protocols within 24 h of collection as previously described.^[26]^ Plasma specimens were stored at –80°C and PBMCs were stored in liquid nitrogen for subsequent plasma markers, immunophenoyping, and SNP analyses respectively. Investigators were blinded to participants’ HIV status during the investigations. Written consent for genetic testing was obtained from all patients (IRB No: 35/09).

### 2.2 Genotyping of *TLR4* and *CD14* single-nucleotide polymorphisms

DNA was extracted from frozen PBMCs and the SNPs of interest *TLR4*/*+896 A*>*G* (rs4986790), +*1196 C*>*T* (rs4986791), and *CD14*/−*260 C*>*T* (rs2569190) were genotyped using the MassARRAYiPLEX platform at the Australian Genome Research Facility (AGRF).

### 2.3 Flow cytometry

Surface staining was done on whole-blood specimens^[28]^ on ice for 30 minutes with the following antibodies: CD14-APC, CD16-PE-Cy7, CD38-PE, HLA-DR-FITC, CD11b-PE (all from BD Biosciences, San Jose, CA), CCR2-PE (R&D Systems), and CX3CR1-PE (MBL International, Woburn, MA) or appropriate isotype-matched negative control antibodies. Immunostained samples were washed twice prior to acquisition on an FACS Canto II Immunocytometry system (BD Biosciences) and analyzed using the FACS Diva software. Data were analyzed using FlowJo (v9.3.1 and v10).

### 2.4 Limulus amebocyte assay

LPS levels were determined in plasma diluted to 1:10 using the chromogenic LAL kit (Lonza, Basel, Switzerland). Plasma LPS-binding proteins were heat inactivated (80°C, 10 minutes) prior to LPS analysis.

### 2.5 ELISA

Commercial ELISA kits were used to determine the levels of sCD163 (IQ products, Groningen, Netherlands), neopterin (Screening EIA, Brahms, Berlin, Germany), sCD14, CX3CL1 and CCL2 (all from Quantikine, R&D Systems, Minneapolis, MN) following the manufacturer's instructions.

### 2.6 Statistical analyses

Genotype frequencies were tested for Hardy–Weinberg equilibrium (HWE) by the chi-square test. Differences of characteristics between patients with and without HIV were compared using Mann–Whitney and Pearson chi-square test or Fisher's Exact Test for continuous and categorical variables, respectively. The association between the *TLR4, CD14* SNPs, and monocyte surface expression of CCR2, CXCR1, CD11b, HLA-DR, and CD38 as well as plasma inflammatory markers LPS, hsCRP, fibrinogen, D-dimer, neopterin, sCD14, sCD163, sCCL2, and sCX3CL1 for both HIV-infected patients and healthy controls were analyzed separately using the Mann–Whitney test; differences in levels were considered significant when *P* < 0.05. *P*-values for each individual comparison were adjusted for multiple comparison using the Benjamin–Hochberg adjustment.^[29,30]^ Due to the significant skew in cIMT that was unable to be corrected using standard (log and square-root) transformations, the associations between *TLR4/CD14* SNPs and median cIMT among HIV-infected patients and healthy controls were analyzed using a median quantile regression model. Step-wise forwards selection of candidate explanatory variables was used to derive the adjusted models to avoid over-fitting. A recessive genetic model was chosen as the primary model of analysis for the *CD14* SNP (TT vs CC/CT) as previous studies have reported the association of this SNP with CVD. Given the allele frequencies in our cohort, we chose AA versus AG as the primary model for *TLR4* SNPs as the GG genotype was not found in the current cohort. The final effect size is reported for 10, 100, or 1000 units depending upon the distribution of the explanatory variable being tested. These transformations were made to ensure we were modeling sensible effect sizes. The analyses were performed using SPSS, v20 (Armonk, NY) and GraphPad PRISM, v5.02 (GraphPad, San Diego, CA) software.

## 3 Results

### 3.1 Demographic characteristics

The clinical characteristics of the 47 HIV-infected individuals and 37 HIV negative healthy controls enrolled in this substudy are outlined in Table 1. Although the original cohort enrolled 51 HIV-infected participants and 49 HIV-negative controls, DNA was successfully extracted less frequently in HIV-uninfected compared to HIV-infected participants. All samples were collected at the same time and stored and then thawed in the 1 laboratory so that there are no clear technical explanations for the differences. Furthermore, we also were unable to identify any significant demographic difference between participants from whom DNA was and was not successfully isolated (data not shown).

**Table 1:**
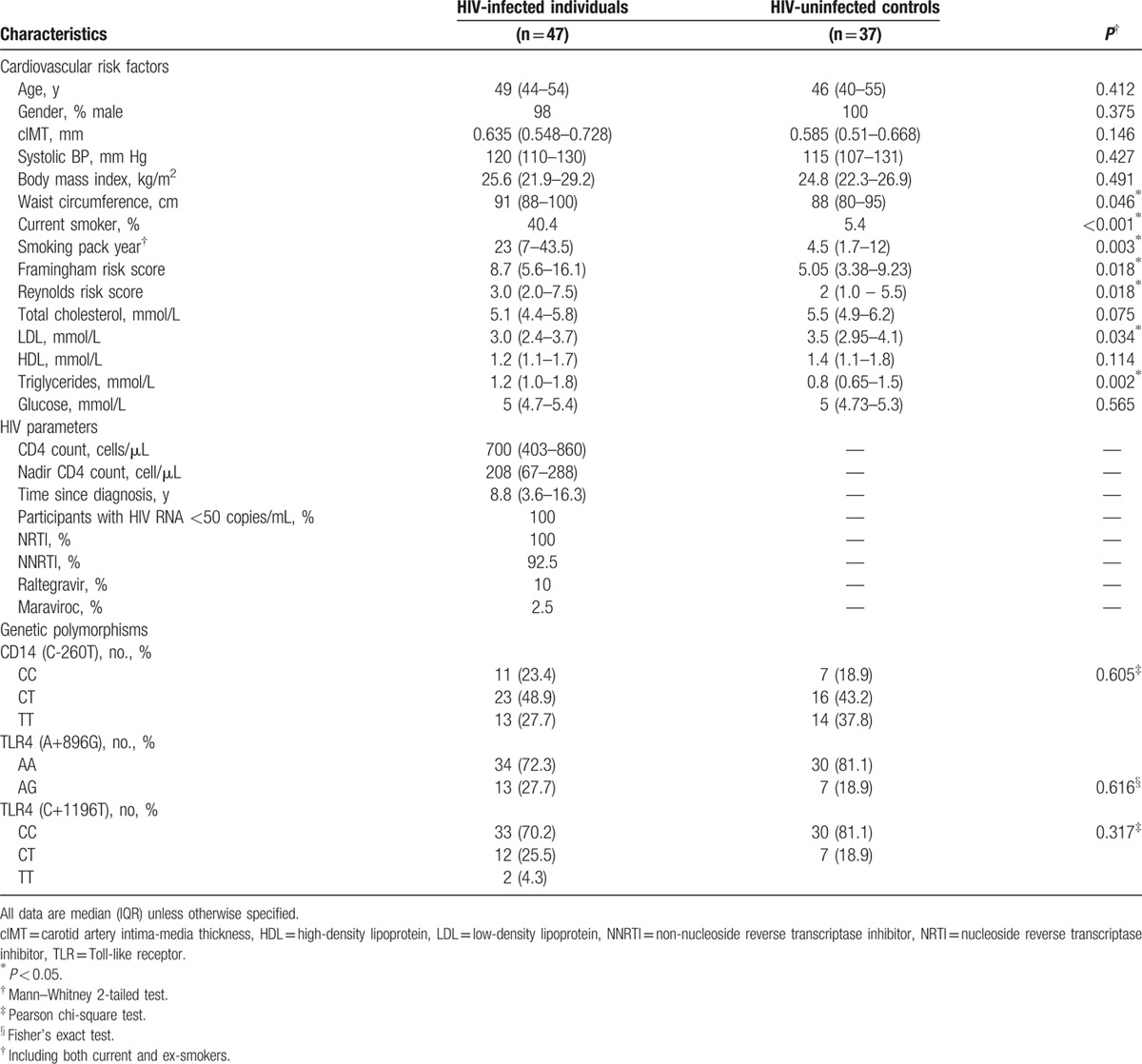
Demographic and clinical characteristics of the study cohort.

The overall study population showed relatively low levels of cardiovascular risk according to traditional risk factor profiles, the Framingham score, although the risk scores were significantly higher in HIV-infected participants compared to HIV-uninfected controls (*P* = 0.018). The median cIMT for HIV-infected participants (0.635 mm) and HIV-uninfected controls (0.585 mm) were both within the healthy range (*P* = 0.146) as reported in the parent study.^[26]^ Subclinical atherosclerosis (defined as median cIMT > 0.7 mm) was identified in 20% of HIV-infected individuals and 10% of healthy controls.

Of note, there were more current smokers in the HIV-infected study group (40.4%) compared to healthy controls (5.4%) (*P* < 0.001) and the median (IQR) of smoking pack years^[31]^ was 23 (7–43.5) in HIV-infected individuals compared to 4.5 (1.7–12) (*P* = 0.003) in healthy controls (Table 1). Although there was no significant difference in cIMT between smokers and nonsmokers, smoking pack years were significantly correlated with cIMT in both HIV-infected individuals and healthy controls (See Supplementary Figure 1).

### 3.2 Allele frequency of TLR4 and CD14 SNPs in HIV-infected and HIV-uninfected participants

Of the 47 HIV-infected participants, 36 carried the T allele *CD14*/−*260* at the *CD14* SNP. Of these 23 were heterozygous (CT) and 13 were homozygous (TT) for *CD14*/−*260* allele. The resulting allele frequency was 0.521 and the distribution was in HWE. The allele frequencies for *TLR4*SNPs +896G and+1196T were 0.138 and 0.170, respectively. The 2 *TLR4* SNPs were in HWE, with the common homozygous genotype co-segregating in 44 of the total 47 individuals (93.6%) studied. There were no participants in the cohort who were homozygous for the GG allele at +896. Two of the HIV-infected participants were homozygous for the TT allele at +1196 (Table 1). The allele frequencies were similar in the HIV-uninfected controls (n = 37) consistent with other studies^[32,33]^ and the HapMap database.

### 3.3 Relationship between *CD14* and *TLR4* genotype and expression of surface markers on monocytes

**Figure 1:**
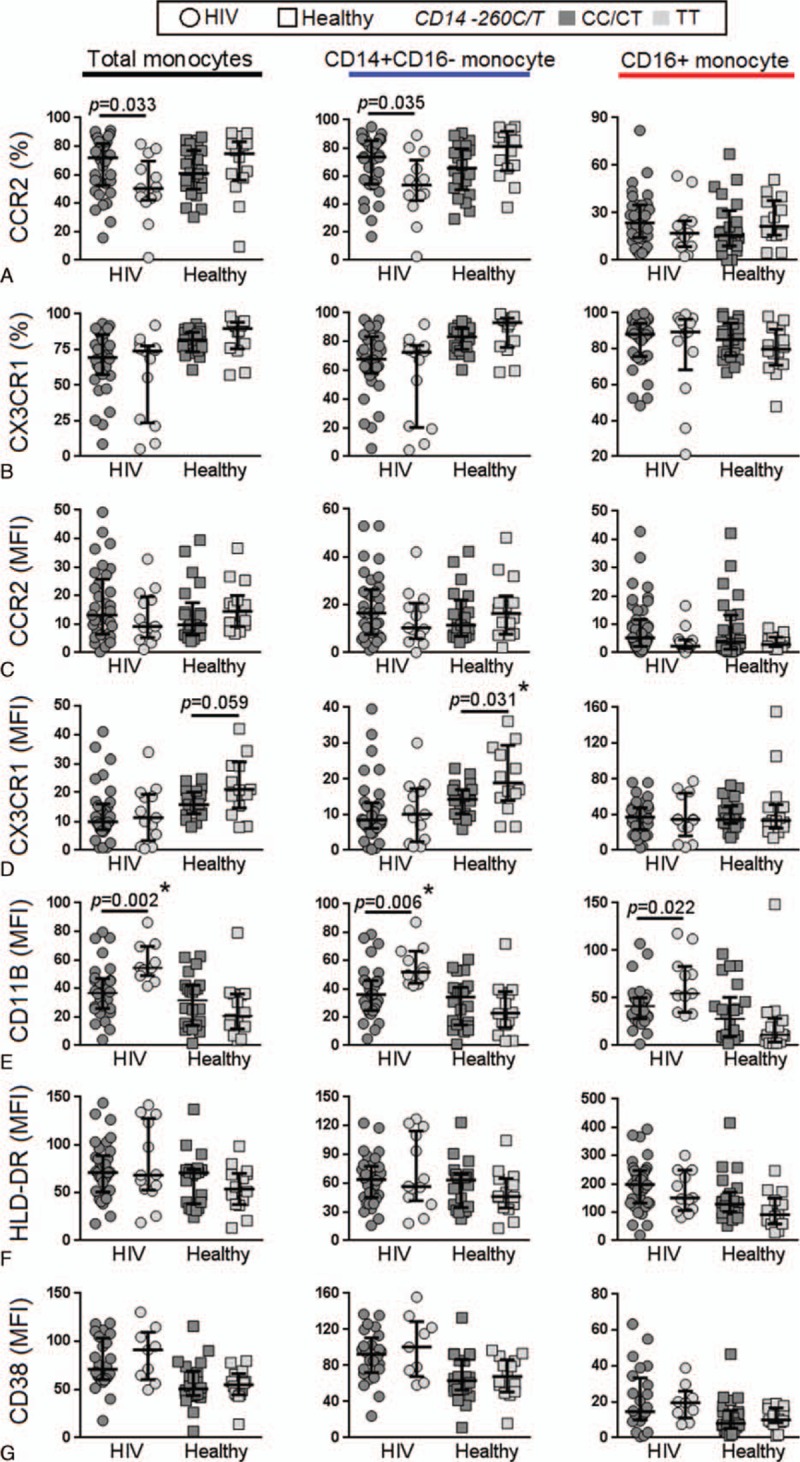
Relationship between the *CD14* (C-260T) genotype and expression of surface markers on monocytes. Expression of various monocytes surface markers were compared between carriers of the *CD14* CC/CT and TT genotypes in HIV-infected and HIV-uninfected (healthy) controls. *P* < 0.05 by the Mann–Whitney test are indicated, with other comparison *P* > 0.05; ^∗^ indicate statistical significant after Benjamin–Hochberg adjustment. HIV = human immunodeficiency virus, MFI = mean fluorescence intensity.

**Figure 2:**
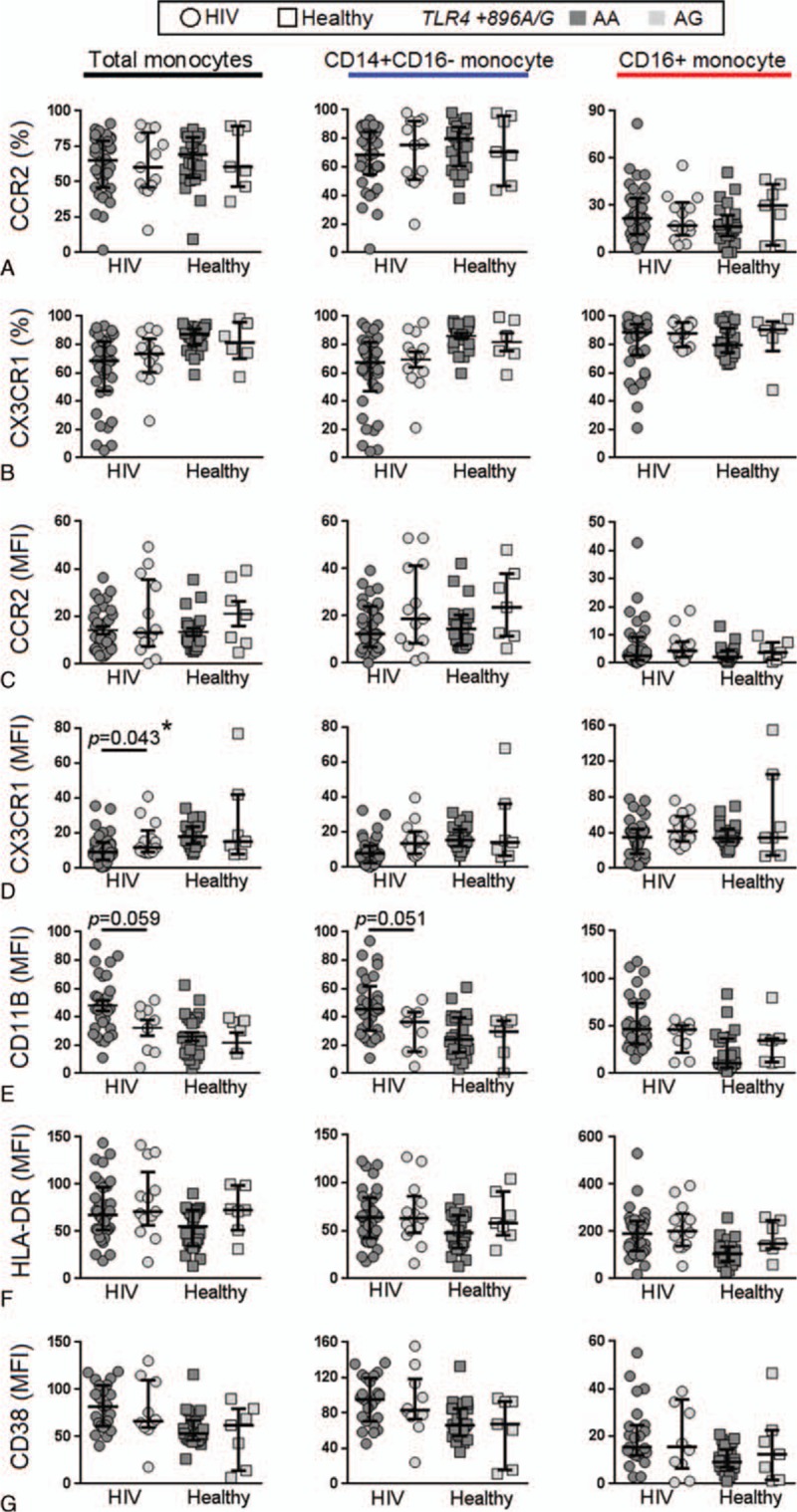
Relationship between the TLR4 (A+896G) genotype and expression of surface markers on monocytes. Expression of various monocytes surface markers were compared between carriers of the *TLR4* AA and AG genotypes in HIV-infected and HIV-uninfected (healthy) controls. *P* < 0.05 by the Mann–Whitney test are indicated, with other comparison *P* > 0.05; ^∗^ indicate statistical significant after Benjamin–Hochberg adjustment.

We compared the expression levels of monocyte activation markers between the*CD14*/−260 TT versus CC/CT genotypes in both HIV-infected and HIV-uninfected individuals (Supplementary Figure 2). Details of the gating strategy used in this study have been previously described.^[26]^ The TT genotype compared to the CC/CT genotype in HIV-infected individuals was associated with reduced expression (percentage) of CCR2 on the total monocyte population (TT = 50% vs CC/CT = 71.8%) and CD14+CD16- monocytes (TT = 56% vs CC/CT = 76.5%) (Fig. 1A) and an increased expression of CD11b on total monocytes (TT = 54.4 vs CC/CT = 37.9%) (measured as mean fluorescence intensity, MFI), CD14+CD16- monocytes (TT = 52 vs CC/CT = 36) and CD16+monocyte (TT = 54.6 vs CC/CT = 41.4) (Fig. 1E). In the HIV-uninfected controls, the TT genotype was only associated with higher expression of CX3CR1 on CD14+CD16- monocytes (TT = 18.9 vs CC/CT 14.3) (Fig. 1D). After Benjamin–Hochberg adjustment for multiple comparisons, only expression of CD11b in total monocytes and CD14+CD16- monocytes in HIV-infected participants and CX3CR1 (MFI) in CD14+CD16- monocytes in healthy controls remained significantly associated with *CD14* −*260* SNP. In addition, as *CD14*/−260 SNP can influence the expression of CD14, we also examined the surface levels of CD14 on monocytes and found no significant difference between the genotypes (Supplementary Figure 3). The *TLR4*/*+896* AG genotype, compared to the AA genotype in HIV-infected participants, was associated with increased expression (MFI) of CX3CR1 in total monocytes (AG = 15 vs AA = 9.3) (Fig. 2D) and a trend toward reduction in expression of CD11b on total monocytes and CD14+ monocytes (Fig. 2E). After Benjamin–Hochberg adjustment, only CX3CR1 (MFI) in total monocytes of HIV-infected participants was associated with *TLR4/+896* SNP. The percentage of CCR2 was not associated with TLR4/+896 SNP and the percentage of CX3CR1 (Fig. 2A) and expression of CCR2, HLA-DR, and CD38 were not associated with both *CD14*/−*260* and *TLR4/+896* SNPS (Fig. 1B, C, F, G and Fig. 2B, C, F, and 2G).

### 3.4 Relationship between *CD14* and *TLR4* genotype and soluble markers of inflammation

**Figure 3:**
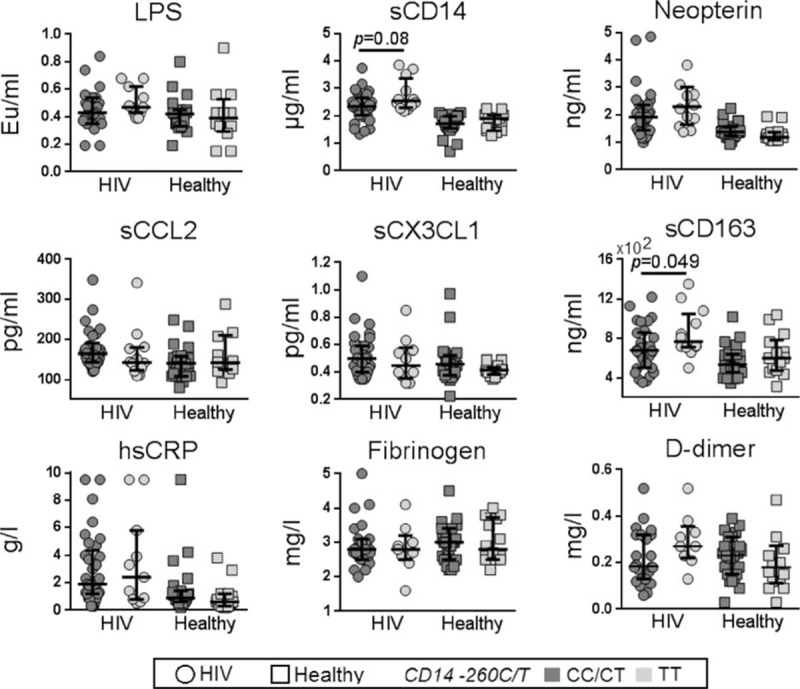
Relationship between the *CD14* (C-260T) genotype and plasma inflammatory markers. The levels of various plasma inflammatory markers were compared between carriers of the *CD14 *CC/CT and TT genotypes in HIV-infected and HIV-uninfected (healthy) controls. *P* < 0.05 by the Mann–Whitney test are indicated, with other comparison *P* > 0.05; ^∗^ indicate statistical significant after Benjamin–Hochberg adjustment. HIV = human immunodeficiency virus, MFI = mean fluorescence intensity.

**Figure 4:**
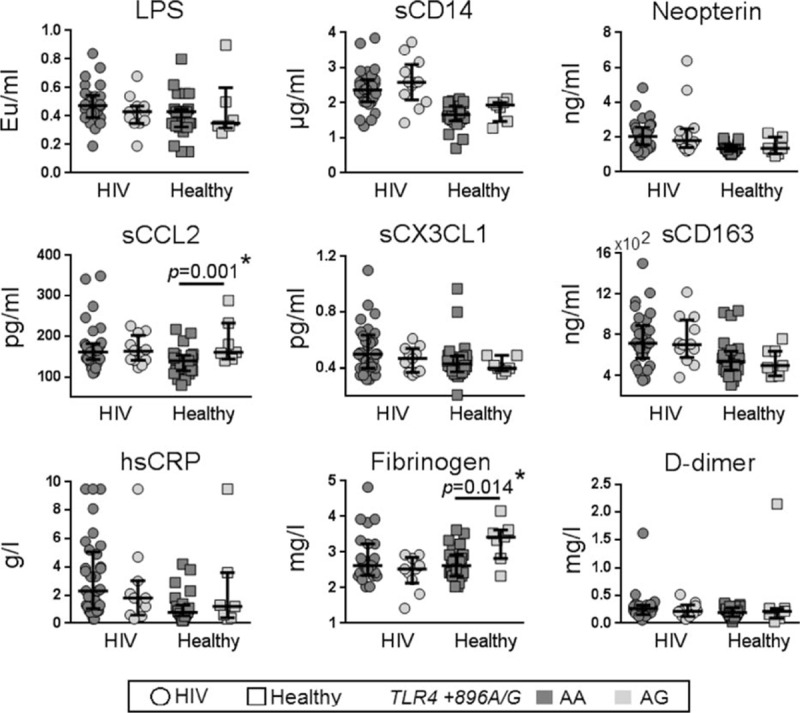
Relationship between the *TLR4* (A+896G) genotype and plasma inflammatory markers. The levels of various plasma inflammatory markers were compared between carriers of the *TLR4* AA and AG genotypes in HIV-infected and HIV-uninfected (healthy) controls. *P* < 0.05 by the Mann–Whitney test are indicated, with other comparison *P* > 0.05; ^∗^ indicate statistical significant after Benjamin–Hochberg adjustment. HIV = human immunodeficiency virus.

Next, we analyzed the association between plasma markers of systemic inflammation and the *CD14*/−*260* TT and CC/CT genotypes (Fig. 3). In HIV-infected patients, we found that the TT genotype was significantly associated with increased levels of sCD163 (median TT = 765.9 pg/mL vs CC/CT = 655.8 pg/mL; *P* = 0.049) and showed a trend of association with higher sCD14 (*P* = 0.08). Given that the level of sCD14 can vary depending on the levels of its stimulant (LPS), we assessed the relationship between *CD14*/−*260* TT and sCD14 using a multiple linear regression model adjusted for plasma LPS. We found that the TT genotype was significantly associated with increased sCD14 (Coef. = 0.233, 95% CI = 0.03–0.44; *P* = 0.026). We observed a decrease in plasma neopterin levels in HIV-uninfected controls with the TT genotype compared to the CC/CT genotypes (*P* = 0.04). There were no other significant differences observed between the CC/CT and TT genotypes with other soluble plasma inflammatory markers investigated. There were no significant associations between *TLR4/+896* AG with any plasma markers among HIV-infected participants but with increased sCCL2 (*P* = 0.01) and fibrinogen (*P* = 0.014) levels in HIV-uninfected controls. After Benjamin–Hochberg adjustment, only sCCL2 and fibrinogen levels in of HIV-infected participants were associated with *TLR4/+896* SNP (Fig. 4).

### 3.5 Relationship between *TLR4* and *CD14* genotype and cIMT

To examine the relationship between *CD14* and *TLR4* SNPs and cIMT, we performed a univariate regression analysis combining both HIV-infected individuals and uninfected controls (n = 84). The monocyte surface and plasma inflammatory markers that showed a trend toward statistical significance using a univariate model were included in a multivariate analysis. The small sample size limited the number of concurrent predictors to avoid over-fitting in the multivariate model. Therefore, a step-wise (forward selection) approach was adopted to derive the multivariate model by including HIV status, Framingham risk score, and CD14 genotype.

As expected, the univariate model showed that several classical risk factors for CVD were significantly associated with cIMT namely age, BMI, HDL, as well as Framingham and Reynolds risk scores (both, *P* < 0.001). We also found a direct association between cIMT and plasma fibrinogen levels (*P* = 0.04) (Table 2). Monocyte markers that were significantly associated with cIMT included the MFI for CD11b expression in total and CD14+ monocytes and HLA-DR expression in CD16+ monocytes, whereas HIV status showed only a trend toward significance (*P* = 0.08). In multivariate modeling adjusting for HIV positive status, Framingham risk score, CX3CR1 expression on pro-inflammatory (CD16+) monocytes and CD14 genotype, only Framingham risk score and CX3CR1 expression on the CD16+ subset of monocytes remained significant. Neither the CD14 nor TLR4 genotypes examined in this analysis were significantly associated with cIMT in either a univariate or multivariate analysis (Table 2).

**Table 2:**
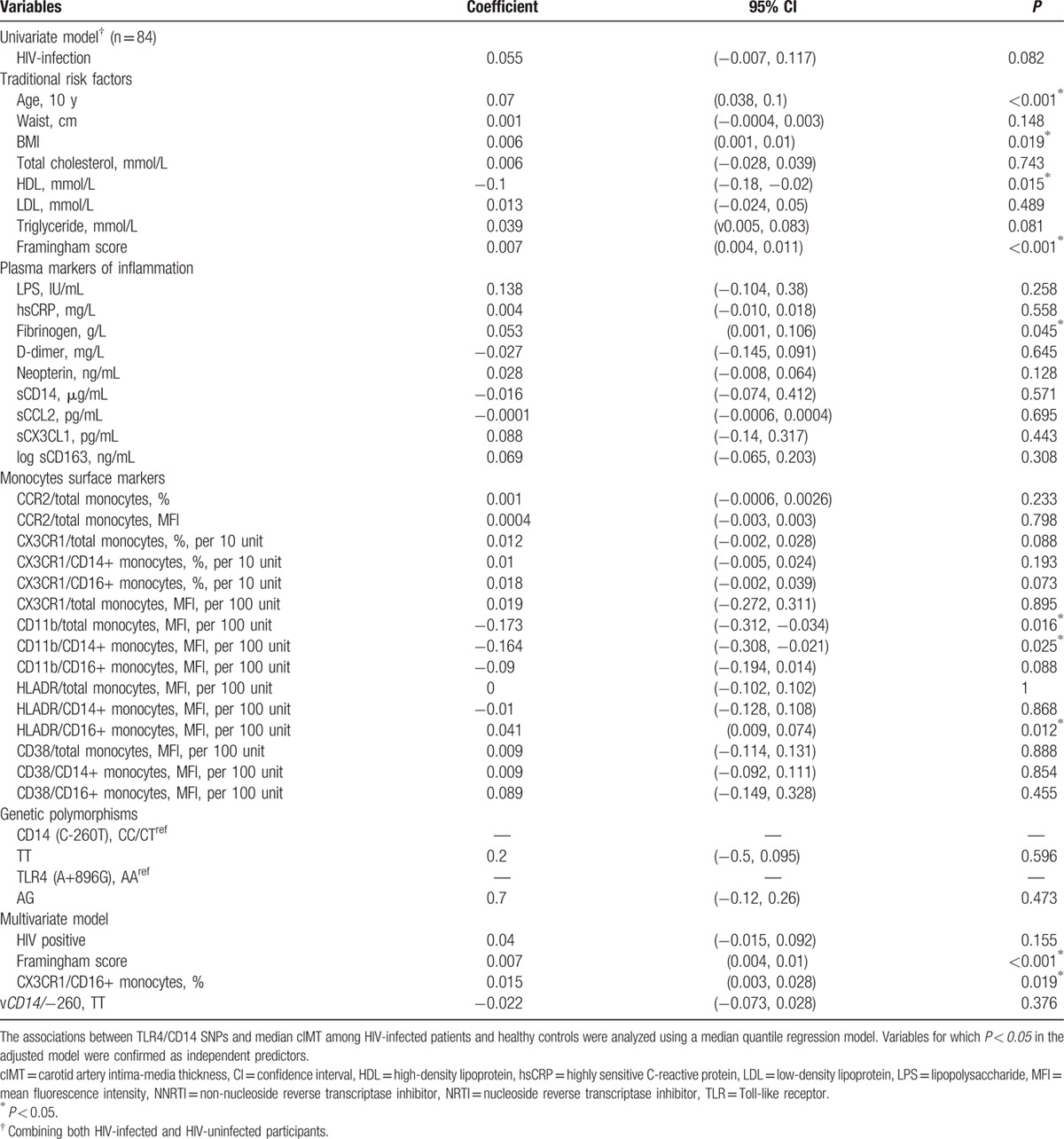
Univariate and multivariate modeling of associations between candidate predictors and cIMT in all participants.

We next performed a multivariable regression model and in a subanalysis of HIV-infected patients (n = 47) the Framingham risk score, percentage of CX3CR1/CD16+ monocyte, MFI of CD11b/total monocyte as well as the TT genotype for *CD14*/−*260* were significantly associated with cIMT at a univariate level. In a multivariate analysis, we adjusted the model for 1 clinical entity only—Framingham score. Being a composite variable, we were unable to adjust for both Framingham and the individual components concurrently in the 1 model secondary to double counting. We preferred Framingham in this case for its superior predictive performance cIMT^[27,34,35]^ and to avoid overfitting the model over a relatively smaller sample size. Controlling for Framingham risk score and percentage of CX3CR1/CD16+ monocytes, we found that the TT genotype was independently associated with decreased cIMT (coef. = –0.054; 95% CI = –0.1, –0.0075; *P* = 0.02) (Table 3).

**Table 3:**
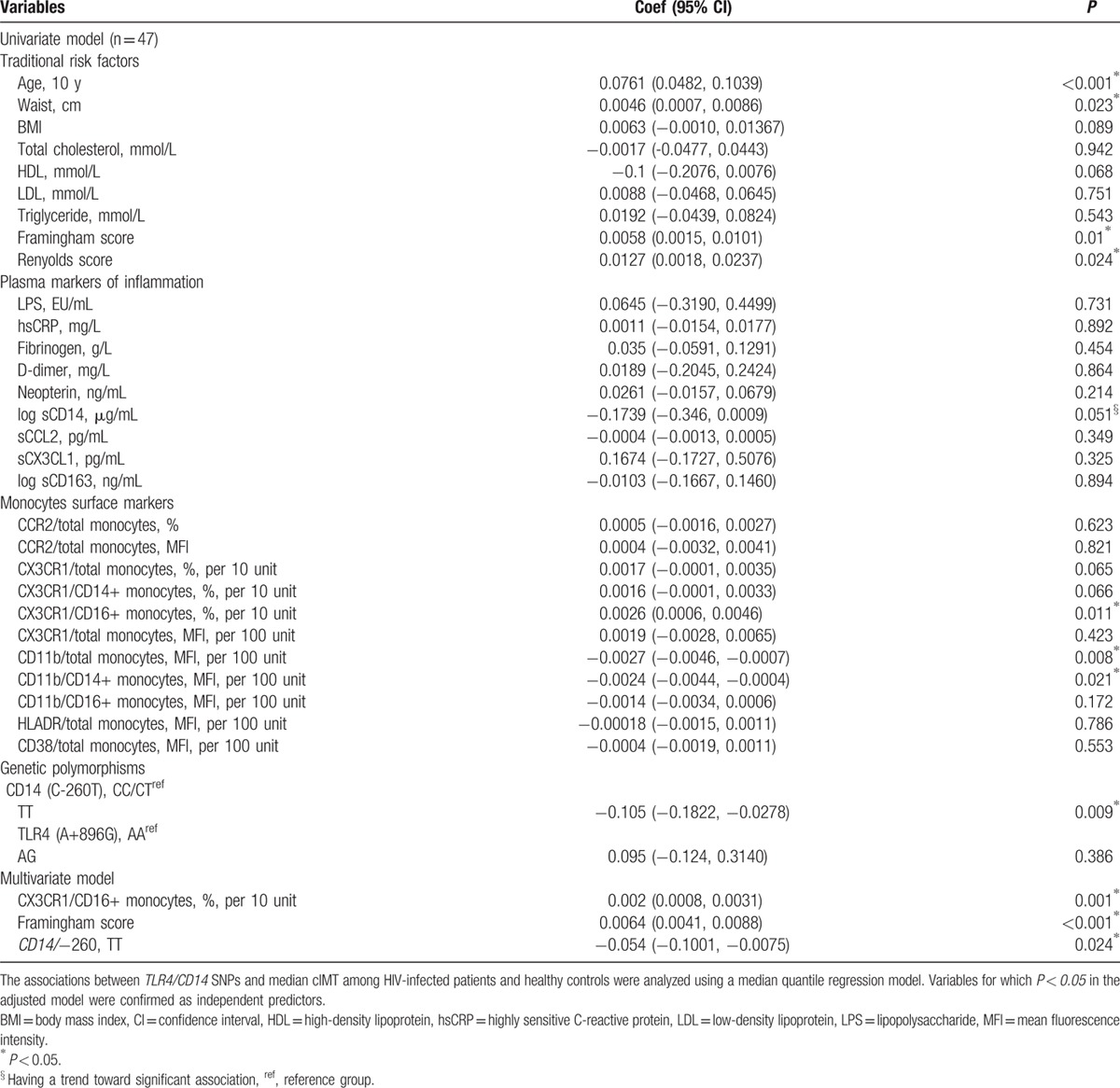
Univariate and multivariate modeling of associations between candidate predictors and cIMT in HIV-infected participants only.

## 4 Discussion

Given the increased risk of CVD in HIV-infected patients on ART, we explored the relationship between SNPs in the CD14 and TLR4 genes, markers of monocyte activation, and cIMT in a Caucasian cohort of HIV-infected patients on ART and HIV-uninfected controls. In HIV-infected patients on ART, we found that the *CD14*/−260 TT genotype was associated with a decreased frequency of CCR2+/CD14+ monocytes and an increased frequency of CD11b+/CD14+ monocytes. The same genotype was also associated with increased levels of sCD14 (after controlling for LPS) among HIV-infected participants. These results suggest an important role of monocyte activation in the progression of CVD in HIV-infected individuals. In an analysis of all participants together, we did not find a significant association between the *CD14 −260* SNP and HIV status with cIMT. However, when HIV-infected individuals were analyzed separately, the TT genotype was associated with reduced cIMT.

Previous reports have shown that *CD14 −260* TT is associated with increased risk of CVD^[20,21,36,37]^ and higher chronic immune activation in some,^[23–25]^ but not all studies.^[22,38]^ In 1 case-control study, individuals with a CC and/or CT genotype were over-represented among participants with CVD and under-represented in healthy controls.^[39]^ In another study involving 450 participants, the CC and CT genotype together with chronic *Chlamydia pneumoniae* infection was significantly associated with the onset of ischemic stroke.^[40]^ These studies are in line with our finding that the CC/CT carriers are associated with increased cIMT as compared to TT. Taken together, it is possible that additional factors—such as chronic elevation of LPS or co-infection—may alter the association of the *CD14 −260* SNP with CVD.

We have also recently examined the relationship of these same polymorphisms with cIMT in a cohort of individuals on ART recruited in Malaysia. We found that the *CD14*/−260 SNP was associated with increased monocyte activation but not with cIMT in the Malaysian cohort.^[25]^ The lack of association between the SNP and cIMT may, in part, be due to the small sample size and low CVD risk (median Framingham risk score = 5) compared to the Caucasian cohort. Another potential difference could be that in the Malaysian cohort, there was a diverse ethnicity including Malay, Chinese, and Indian.

Atherosclerosis is widely regarded as a chronic inflammatory disease and atherosclerotic lesions have been shown to contain large numbers of immune cells, particularly macrophages and T-cells.^[41]^ Atherosclerotic lesions often begin with activation of endothelial cells that line the wall of blood vessels,^[42]^ a process promoted by HIV infection.^[43]^ Upon activation, the endothelial cells start to recruit circulating monocytes that migrate into the sub-endothelial lining. These monocytes will then differentiate into macrophages and release pro-inflammatory cytokines and recruit more monocytes to the lesion.^[41,43]^

In HIV-infected individuals in this study, we found that the *CD14*/−260 TT genotype was associated with lower CCR2 and higher CD11b expression on total and CD14+ monocytes. The chemokine CCL2 (monocyte chemoattractant protein-1/MCP-1) is highly expressed in human atherosclerotic plaques.^[44]^ CCR2 is the receptor for CCL2 expressing on monocytes,^[45]^ and binding to this receptor facilitates the recruitment of monocytes into the subendothelial spaces of atherosclerotic lesions.^[46]^ The association between *CD14*/−*260* TT genotype and low CCR2 expression may explain the association between the TT genotype and reduced cIMT among HIV-infected individuals.

In a mouse model, CD11b+ monocytes have been found to preferentially adhere and migrate into atherogenicplaques.^[47]^ However, in CD11b^−/−^ and CD11b^+/+^ transgenic mice that were fed with a high-fat diet for 16 weeks, CD11b was not associated with artherogenesis.^[48]^ CD11b has also been known to mediate fibrin degradation.^[49]^ The binding of fibrinogen to the surface integrin Mac-1 (CD11b/CD18) on monocytes allows internalization of the complex into lysosomes for degradation by aspartyl protease cathepsin D.^[49]^ Finally, CD11b expressing monocytes areinvolved in resolution of inflammation.^[50]^ Therefore, the presence of CD11b+ monocytes in atherosclerotic lesions could be physiologic, perhaps even having a protective, or reparative, rather than plaque-forming function.^[51]^ Here we found an association between *CD14*/−*260* TT genotype and high CD11b expression, which again may have contributed to reduced cIMT among HIV-infected individuals.

*CD14 −260 TT* has been shown to enhance the expression of CD14.^[17,19]^ Here did not find this association but found an association with elevated levels of sCD14 (after adjusting for LPS), consistent with findings from a Malaysia cohort that we recently reported.^[25,52]^ CD14 is the receptor for LPS and is shed from the monocyte surface following proinflammatory stimulation by LPS.^[53]^ This may potentially be occurring at a higher frequency in HIV-infected individuals given the high persistent levels of LPS and may explain why we found an association between the TT genotype in this population with levels of sCD14 but not expression of CD14 on monocytes.

Interestingly, the relationship between *CD14/−260* TT genotypes and surface and soluble markers of monocyte activation as well as cIMT differed between the HIV-infected individuals and uninfected controls. This may potentially be explained by higher levels of microbial translocation and chronic immune activation that persists despite ART among HIV-infected individuals.^[54]^ Therefore, the potential influence of this SNP could be greater in HIV-infected individuals due to higher levels of microbial translocation and immune activation.

In this same cohort, we previously demonstrated using multivariate modeling that the expression of CD11b and CX3CR1 were independent predictors of cIMT.^[26]^ In the current study, we showed that the *CD14/−260* SNP was significantly associated with cIMT in HIV-infected individuals, but this was independent from the expression of CX3CR1 and traditional risk factors, that is, Framingham risk score. The *CD14/−260*SNP, however, was also strongly associated with CD11bexpression; therefore, we only included*CD14/−260* in the regression analysis as inclusion of 1 variable will effectively take account of the other variable.

In the current study, we surprisingly did not find an association between HIV and CVD as reported by previous studies.^[1,55–57]^ This may be due to differences in clinical endpoints measured in different studies as previous studies examined individuals with a clinical endpoint,^[1,58,59]^ while here we measured a subclinical parameter of cIMT. Furthermore, only 20% of HIV-infected patients in this study had evidence of subclinical atherosclerosis. However, the HIV-infected individuals compared to uninfected individuals had a higher Framingham risk score.

Our current study has some limitations. First, the sample size was small, which limited the number of variables we could include in a multivariable analysis. Second, the HIV-infected and HIV-uninfected individuals had some significant differences in relation to CVD risk and specifically the number of participants who smoked and their smoking pack years, although this difference was controlled in multivariate analyses incorporating the Framingham score. Finally, we only assessed cIMT and ideally a study that uses a clinical endpoint would be preferable although this would mean a very substantial increase in study sample size.

In conclusion, SNPs in *CD14/−260* and *TLR4/+896* were significantly associated with different markers of systemic and monocyte activation and cIMT that differed between HIV-infected participants on ART and HIV-uninfected controls. Further investigation on the relationship of these SNPs with a clinical endpoint of CVD is warranted in HIV-infected patients on ART.

## Supplementary Material

Supplemental Digital Content
