# Supplementary material for: Genetic polymorphisms in the CD14 gene are associated with monocyte activation and carotid intima-media thickness in HIV-infected patients on antiretroviral therapy
Source: Medicine (Baltimore). 2016 Aug 7;95(31):e4477. doi: 10.1097/MD.0000000000004477 (PMC4979844; doi:10.1097/MD.0000000000004477)
Supplement: Supplemental Digital Content [file medi-95-e4477-s001.doc]

**
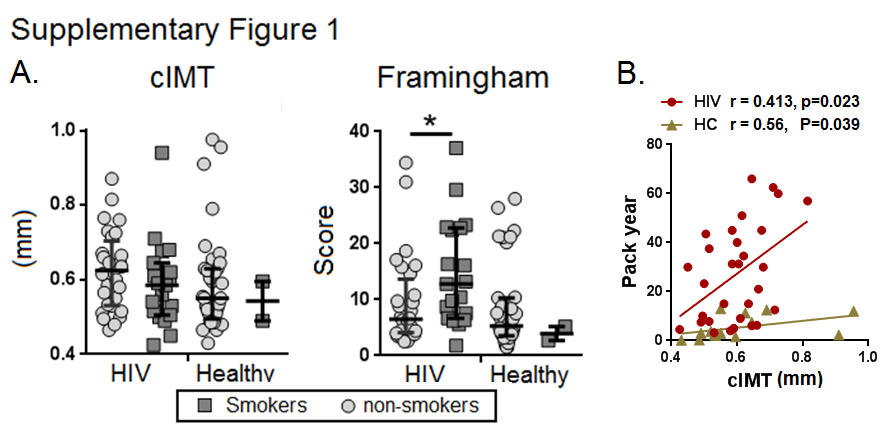
**

**Supplementary Figure 1. Relationship between HIV, smoking, Framingham score and cIMT. A.** cIMT (left) and Framingham scores (right) for HIV-infected participants and health controls who were current smokers (squares) and non-smokers (circles). *, *p*<0.05 by Mann-Whitney U test. **B.** Relationship between smoking pack years and median cIMT for smokers who were HIV-infected (red circles) and healthy controls (brown triangles)., Spearman correlation co-efficients and p values are shown.

**
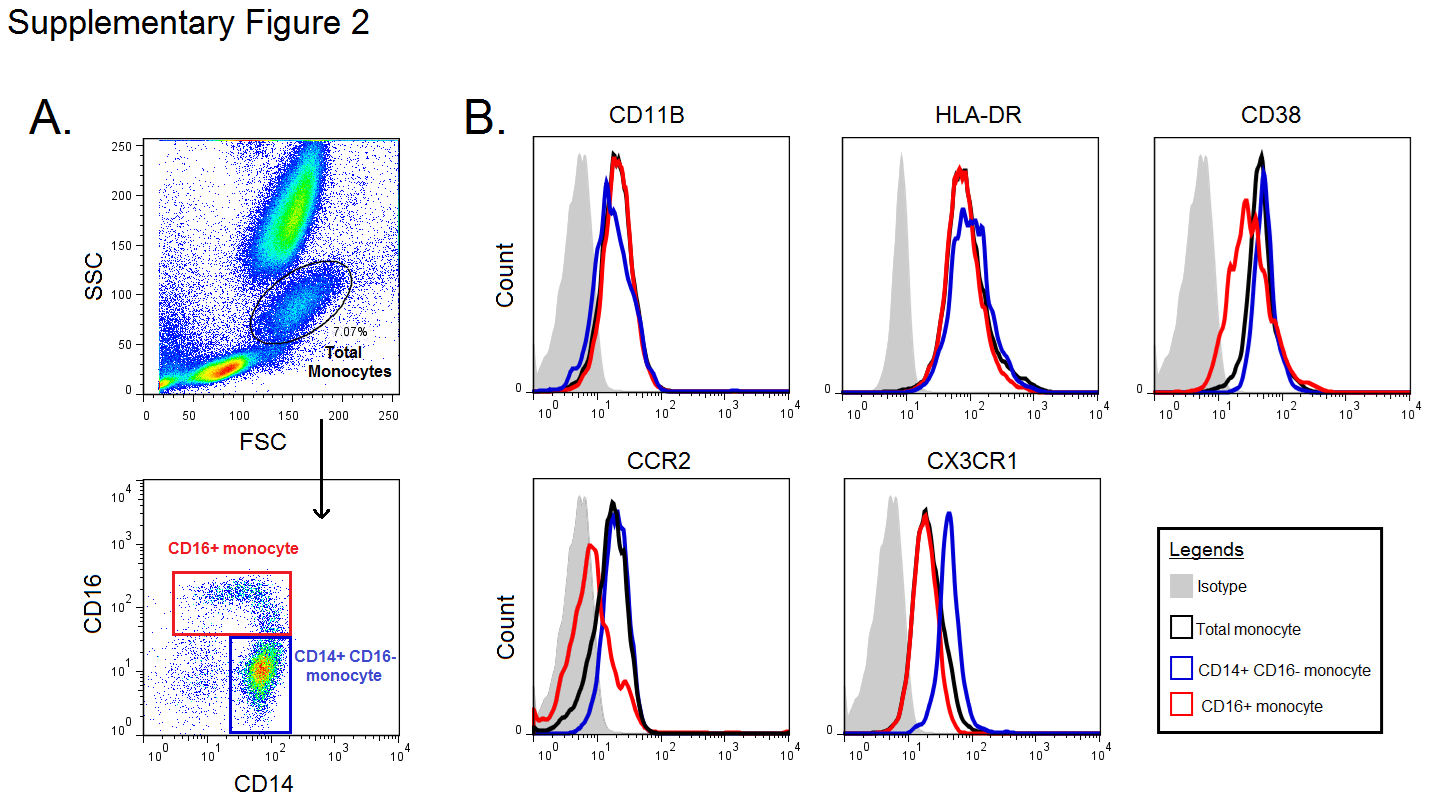
**

**Supplementary Figure 2. Subset of circulating monocyte and expression of surface markers.** (**A**) Representative flow cytometry plots showing total monocytes and the distribution of the monocyte subsets defined by CD14 and CD16 markers i.e. CD14++CD16- and CD16+monocytes. (**B**) Mean fluorescence intensity of expression of CD11B, HLA-DR, CD38, CCR2 and CX3CR1 in monocyte subsets are shown as histograms.

**
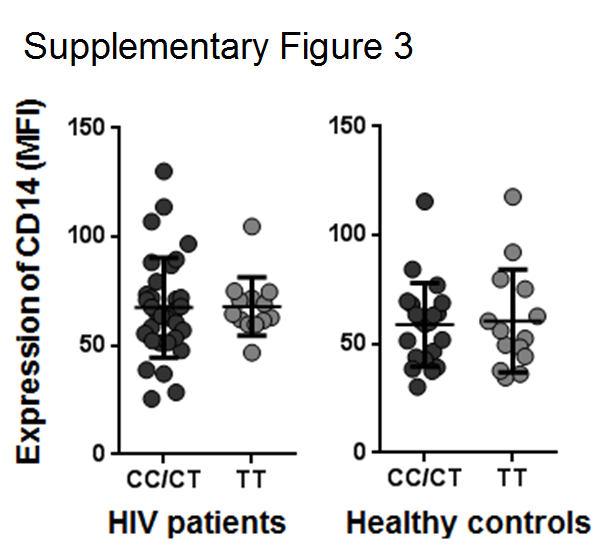
**

**Supplementary Figure 32. Relationship between the *CD14* (C-260T) genotype and surface expression of CD14 on monocytes.** Expression of membrane bound CD14 were compared between carriers of the *CD14* CC/CT and TT genotypes in HIV-infected and HIV-uninfected (healthy) controls. *, *p*<0.05, with other comparison *p*>0.05; MFI, mean fluorescence intensity.
